# Comparative Effects of *Trichoderma guizhouense* NJAU4742 and *Bacillus velezensis* SQR9 on Growth and Pb Accumulation in *Salix suchowensis*

**DOI:** 10.3390/ijms26209961

**Published:** 2025-10-13

**Authors:** Ruifang Huang, Baosong Wang, Ming Xu, Dezong Sui, Xudong He

**Affiliations:** 1 Jiangsu Academy of Forestry, Nanjing 211153, China; aion126@126.com (R.H.); jsaf_wbs@outlook.com (B.W.); xuming2009@126.com (M.X.); suidezong@163.com (D.S.); 2 The Jiangsu Provincial Infrastructure for Conservation and Utilization of Agricultural Germplasm, Nanjing 210014, China

**Keywords:** *S. suchowensis*, Pb contamination, phytoremediation, microbial inoculants, plant growth promotion

## Abstract

Soil lead (Pb) contamination poses a severe threat to agricultural sustainability and food security. Phytoremediation offers a green alternative for remediation, yet its efficiency is limited by poor plant tolerance and restricted metal uptake. In this study we investigated the functional roles of the microbial inoculants *Trichoderma guizhouense* NJAU4742 and *Bacillus velezensis* SQR9 in enhancing the performance of *Salix suchowensis* P1024 grown in Pb-contaminated soil. NJAU4742 significantly increased plant biomass by 34% (*p* < 0.05), accompanied by increased soil microbial biomass and higher activities of urease, acid phosphatase, and sucrase. In contrast, SQR9 strongly enhanced Pb accumulation by 19% (*p* < 0.05), which was accompanied by upregulated antioxidant enzymes, reduced lipid peroxidation, and elevated cysteine levels. Random forest and correlation analyses demonstrated that soil nutrient cycling indices (urease, MBC, sucrase) were key predictors of biomass, whereas antioxidant defenses (POD, CAT) primarily explained Pb accumulation. These findings provide new insights into the distinct contributions of NJAU4742 and SQR9 to willow growth and Pb remediation, and provide a basis for developing more effective microbe-assisted phytoremediation strategies.

## 1. Introduction

Heavy metal contamination has become a critical environmental issue worldwide due to rapid industrialization, urbanization, and intensive agricultural activities [1]. Among heavy metals, Pb is one of the most widespread and persistent pollutants, largely released from mining, smelting, battery manufacturing, and traffic emissions [2]. Elevated Pb concentrations can impair soil quality, suppress microbial activity, reduce crop yields, and threaten public health through dietary exposure [3]. In China alone, Pb-contaminated farmlands are widely distributed in industrialized regions, severely restricting safe agricultural production and sustainable land use. Therefore, the development of cost-effective, environmentally friendly strategies for Pb remediation is an urgent global priority.

Conventional remediation methods, such as excavation, soil washing, and chemical stabilization, are often unsuitable for large-scale applications because they are costly and disruptive. In contrast, phytoremediation utilizes plants to extract, stabilize, or transform contaminants, offering advantages such as low cost, ecological compatibility, and potential biomass utilization [4,5]. However, phytoremediation efficiency is often constrained by two major challenges: (i) poor plant growth under heavy metal stress due to toxicity-induced inhibition of physiological processes, and (ii) limited metal uptake or sequestration capacity in many candidate species [6]. In this context, plant–microbe partnerships have emerged as a promising strategy. Beneficial microorganisms can colonize the rhizosphere or plant tissues and provide multiple benefits that mitigate heavy metal stress [7]. These include enhancing nutrient availability, secreting organic acids and siderophores that alter metal mobility, producing phytohormones that stimulate plant growth, and activating antioxidant systems that alleviate oxidative damage [8,9]. Consequently, microbial inoculants are increasingly recognized as effective biofertilizers for phytoremediation.

Recent studies provide strong evidence that microbial inoculation can simultaneously promote metal absorption and stimulate plant growth, thereby improving phytoremediation efficiency. For example, inoculation of *Phytolacca acinosa* with the endophyte *Bacillus cereus* PE31 enhanced Cd uptake by more than 50% while also increasing plant biomass by 28–31%, through elevating rhizosphere Cd and phosphorus bioavailability and improving nutrient status [10]. Similarly, co-inoculation of *Phytolacca* species with *Lactobacillus* strains further improved Cd remediation efficiency and plant vigor by modulating rhizosphere chemistry and facilitating nutrient–metal interactions [1]. In another study, inoculation of the hyperaccumulator *Sedum alfredii* with multi-metal-resistant *Bacillus subtilis* strains increased shoot accumulation of Cd, Zn, Cu, and Pb by up to 90%, while also enhancing biomass production, partly due to rhizosphere acidification, siderophore secretion, and upregulation of metal transporter genes [11]. These findings underscore the capacity of beneficial microorganisms to address key constraints of conventional phytoremediation and to substantially enhance its effectiveness. *Trichoderma guizhouense* NJAU4742 and *Bacillus velezensis* SQR9 were identified as promising microbial inoculants by our collaborator’s laboratory previously. *T. guizhouense* NJAU4742 has been reported as a highly rhizosphere-competent strain capable of secreting abundant indole-3-acetic acid, cellulase, and β-1,3-glucanase, thereby promoting root development and enhancing nutrient cycling under stress conditions [12,13]. In contrast, *B. velezensis* SQR9 is a well-characterized plant growth–promoting rhizobacterium known for its strong biofilm-forming ability, secretion of organic acids and siderophores, and induction of plant systemic resistance [14,15]. However, their specific roles in Pb-contaminated systems remain poorly understood, and few studies have directly examined how these two microbes may differentially influence plant growth and Pb uptake.

Willow (*Salix* spp.) has been widely recognized as a promising phytoremediation species due to its rapid growth, extensive root system, high biomass yield, and strong tolerance to heavy metals [16]. However, its performance under severe Pb stress is often limited by growth inhibition and oxidative damage, reducing its remediation efficiency [17]. Introducing beneficial microbial inoculants may therefore provide a practical means of overcoming these barriers by simultaneously enhancing willow growth and Pb tolerance. In this study, we selected *S. suchowensis* P1024, a clonal line previously screened for its high Pb remediation potential. Multiple levels of analysis, encompassing plant physiology, biochemistry, and soil ecology, together with advanced statistical approaches, were employed to comprehensively evaluate the treatment effects of *T. guizhouense* NJAU4742 and *B. velezensis* SQR9. We conducted a controlled pot experiment using Pb-contaminated field soil to evaluate (i) plant growth responses and Pb accumulation, (ii) changes in antioxidant enzyme activities and stress-related metabolites, (iii) alterations in soil microbial biomass and enzyme activities, and (iv) integrative statistical relationships between soil and physiological parameters with plant performance. The aim of this work is to clarify the functional roles of *Trichoderma* and *Bacillus* in enhancing willow growth and Pb remediation, thereby providing new insights into strategies for enhancing plant productivity and remediation efficiency in Pb-contaminated soils.

## 2. Results and Discussion

### 2.1. Differential Effects of Microbial Inoculation on Willow Growth and Pb Accumulation

**Figure 1:**
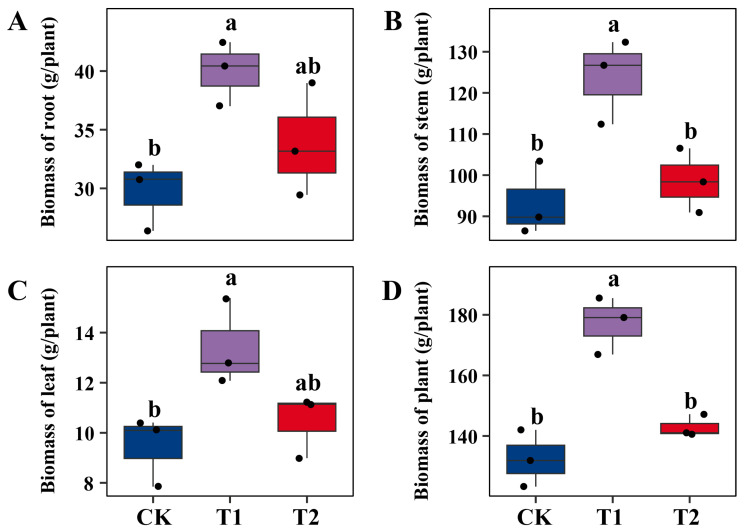
Biomass of *S. suchowensis* P1024 under different treatments in Pb-contaminated soil. Panels (**A**–**D**) represent root, stem, leaf, and total plant biomass, respectively. CK denotes the control treatment with organic fertilizer only, T1 indicates inoculation with *T. guizhouense* NJAU4742, and T2 indicates inoculation with *B. velezensis* SQR9. Each treatment included three biological replicates. Data are shown as boxplots with individual data points. Different lowercase letters above bars indicate significant differences among treatments at *p* < 0.05 according to one-way ANOVA followed by Tukey’s multiple comparison test.

Application of microbial fertilizers altered the biomass of *S. suchowensis* P1024 under Pb stress, with *T. guizhouense* NJAU4742 (T1) exerting stronger effects than *B. velezensis* SQR9 (T2) (Figure 1). Root biomass was increased by approximately 14% under T2 and by 35% under T1, with T1 showing a significant advantage over CK (Figure 1A). For stems and leaves, NJAU4742 consistently promoted greater growth than SQR9, though the magnitude of improvement was less pronounced than in roots (Figure 1B,C). Total biomass reflected the cumulative effects. Compared with CK, T1 enhanced total biomass by nearly 34% with statistically significant increase, whereas T2 improved it only modestly (about 8%) without significance (Figure 1D). Previous studies have shown that *T. guizhouense* NJAU4742 promotes plant growth by secreting phytohormones, producing cell wall–degrading enzymes, and releasing volatile compounds [18,19]. In Pb-contaminated soils, *Trichoderma* also plays an important role in reducing metal toxicity by excreting organic acids and siderophores that immobilize Pb, thereby lowering its bioavailability to plants [20]. These functions not only protect the root system from heavy metal stress but also maintain photosynthetic capacity, resulting in greater biomass accumulation. By contrast, *B. velezensis* SQR9 is widely recognized as a rhizosphere-competent bacterium that promotes biofilm formation, suppresses soilborne pathogens, and produces antimicrobial lipopeptides such as surfactin and fengycin [14,15,21]. Although these traits enhance rhizosphere health and provide indirect growth benefits, their direct impact on biomass accumulation under heavy metal stress appears more limited compared with *Trichoderma*. In the present study, SQR9 contributed to modest improvements in biomass, suggesting that its main role under Pb stress may lie in protecting root systems from pathogen invasion and maintaining rhizosphere stability, rather than directly stimulating vigorous plant growth.

**Figure 2:**
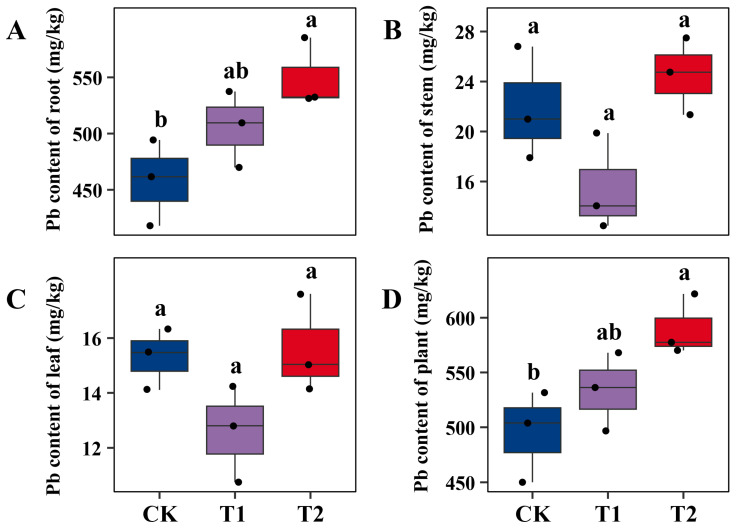
Pb content of *S. suchowensis* P1024 under different treatments in Pb-contaminated soil. Panels (**A**–**D**) represent Pb concentrations in roots, stems, leaves, and whole plants, respectively. CK denotes the control treatment with organic fertilizer only, T1 indicates inoculation with *T. guizhouense* NJAU4742, and T2 indicates inoculation with *B. velezensis* SQR9. Each treatment included three biological replicates. Data are shown as boxplots with individual data points. Different lowercase letters above bars indicate significant differences among treatments at *p* < 0.05 according to one-way ANOVA followed by Tukey’s multiple comparison test.

Microbial fertilizer application also altered the distribution and accumulation of Pb in *S. suchowensis* P1024, with *B. velezensis* SQR9 (T2) exerting stronger effects than *T. guizhouense* NJAU4742 (T1) (Figure 2). Compared with CK, Pb accumulation in roots increased by approximately 10% under T1 and by 20% under T2, with the latter being statistically higher (Figure 2A). This indicates that SQR9 enhanced Pb sequestration in roots, which may be attributed to its strong rhizosphere colonization capacity and ability to influence root physiology and stress responses [14,22]. In stems and leaves, differences among treatments were less pronounced. Stem Pb content varied slightly but did not show significant differences among CK, T1, and T2 (Figure 2B). Similarly, leaf Pb content remained relatively stable across treatments, suggesting that microbial inoculation primarily affected Pb accumulation in belowground tissues rather than in aerial organs (Figure 2C). Total Pb accumulation (Figure 2D) reflected the strong contribution of root sequestration. Compared with CK, T1 increased whole-plant Pb uptake by about 8%, while T2 raised it by nearly 19%. These results demonstrate that SQR9 was more effective than NJAU4742 in enhancing overall Pb accumulation, primarily by promoting Pb retention in roots. As *B. velezensis* strains are known to secrete organic acids and siderophores, these metabolites may contribute to mobilizing heavy metals in the rhizosphere and thereby increase their availability for root uptake [23]. In addition, biofilm formation and secretion of extracellular polymeric substances (EPS) may facilitate Pb immobilization at the root surface, reducing translocation to shoots but enhancing overall Pb sequestration [24,25]. By contrast, *Trichoderma* spp. often promotes biomass accumulation and stress tolerance more strongly than heavy metal uptake. Thus, in this study, NJAU4742 contributed to moderate increases in Pb accumulation, but its effect was less pronounced than that of SQR9. *Bacillus* species are widely recognized for their beneficial roles in metal remediation. Chen et al. [26] reported that inoculation with *Bacillus megaterium* B6 increased Pb transport from roots to branches in *Salix integra* by approximately two-fold. The authors attributed this effect to improved rhizosphere microbial community structure and upregulation of ABC-type transporters that enhanced amino-acid chelation of Pb, thereby reducing its toxicity. These findings align with our observations that *B. velezensis* SQR9 facilitates Pb mobilization and sequestration through both biochemical and physiological pathways. In addition, the Pb-contaminated soil used in this study was collected from the vicinity of a lead–acid battery manufacturing facility, where total Pb concentrations reached approximately 17.55 g·kg^−1^. Although this level is much higher than typical agricultural soils, it reflects the extreme contamination commonly found at industrial legacy sites. Willow species combined with microbial inoculants are well suited for remediating such severely polluted areas, which are usually limited in size. In agricultural soils with moderate Pb levels (generally <0.1 g·kg^−1^), remediation can instead be achieved through agronomic stabilization practices such as lime or biochar amendment, or by using low-cost tolerant plants like *Sedum* or *Phytolacca*.

### 2.2. Enhancement of Antioxidant Defense and Detoxification Pathways by Microbial Inoculation Under Pb Stress

**Figure 3:**
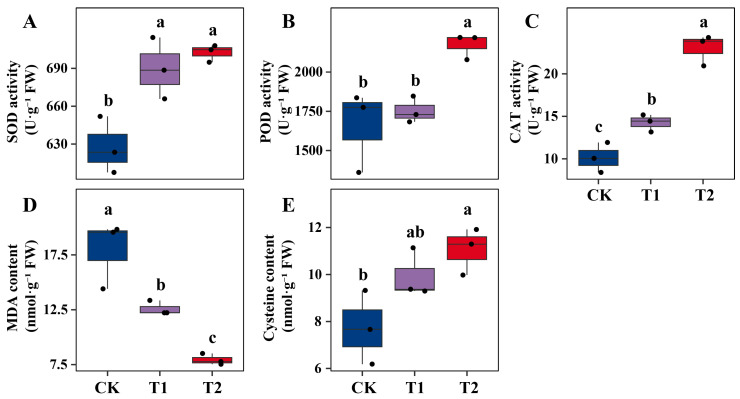
Physiological parameters measured in the leaves of *S. suchowensis* P1024 under different treatments in Pb-contaminated soil. Panels (**A**–**E**) represent superoxide dismutase (SOD), peroxidase (POD), catalase (CAT), malondialdehyde (MDA), and cysteine (Cys), respectively. CK denotes the control treatment with organic fertilizer only, T1 indicates inoculation with *T. guizhouense* NJAU4742, and T2 indicates inoculation with *B. velezensis* SQR9. Each treatment included three biological replicates. Data are shown as boxplots with individual data points. Different lowercase letters above bars indicate significant differences among treatments at *p* < 0.05 according to one-way ANOVA followed by Tukey’s multiple comparison test.

Microbial inoculation significantly modified the physiological status of *S. suchowensis* P1024 under Pb stress, particularly in terms of antioxidant defense and metal detoxification capacity (Figure 3). Both *T. guizhouense* NJAU4742 (T1) and *B. velezensis* SQR9 (T2) enhanced the activities of key antioxidant enzymes, although the magnitude of improvement differed between strains. SOD activity increased significantly under both T1 and T2 compared with CK, with T2 exhibiting the slightly stronger effect (Figure 3A). Elevated SOD activity accelerates the dismutation of superoxide radicals into H_2_O_2_, reducing oxidative injury in Pb-stressed cells [27]. POD activity was also stimulated, with T2 showing a substantially greater increase than T1 (Figure 3B). POD is crucial for scavenging H_2_O_2_ and for reinforcing cell walls through lignin biosynthesis, which may also limit Pb translocation [28]. Notably, CAT activity displayed the most striking enhancement: T1 raised CAT activity moderately, while T2 induced more than a twofold increase over CK (Figure 3C). Since CAT directly decomposes H_2_O_2_ into water and oxygen, the sharp increase under SQR9 inoculation indicates a robust capacity for detoxifying ROS, aligning with previous reports that *Bacillus* strains effectively upregulate host antioxidant enzymes under metal stress [29,30].

MDA content is a marker of lipid peroxidation and membrane damage [31], and was observed declined markedly with microbial treatments. T1 reduced MDA by nearly one-third, while T2 decreased it by more than half relative to CK (Figure 3D). This reduction indicates that microbial inoculation effectively alleviated Pb-induced oxidative stress, with SQR9 conferring the greatest protection. The improvement may be linked to the combined upregulation of SOD, POD, and CAT, which synergistically limit ROS accumulation and lipid peroxidation [32]. Reduced MDA levels signify improved stress tolerance and help preserve membrane integrity, which is essential for maintaining nutrient uptake and growth under contamination stress [33]. Cysteine concentrations were elevated following microbial inoculation, with T2 producing the most significant increase (Figure 3E). As cysteine is the precursor of glutathione (GSH) and phytochelatins (PCs), which are major chelators of heavy metals, higher cysteine availability might indicate an enhanced capacity for Pb chelation, sequestration, and detoxification [34,35,36]. By contrast, NJAU4742 induced moderate increases in cysteine, suggesting a supportive but less dominant role in Pb chelation compared with SQR9. Collectively, these physiological results reveal mechanistic differences between the two inoculants. NJAU4742 primarily promoted biomass accumulation while moderately improving antioxidant defense, indicating its role is more growth-oriented and indirectly protective under Pb stress. In contrast, SQR9 strongly activated antioxidant enzyme systems and reduced oxidative damage, thereby directly strengthening the physiological resilience of willow to Pb toxicity. This functional complementarity provides a rationale for combining the two strains in future applications to simultaneously maximize growth and Pb tolerance.

### 2.3. Stimulation of Soil Microbial Biomass and Enzymatic Activities by Microbial Inoculation Under Pb Stress

**Figure 4:**
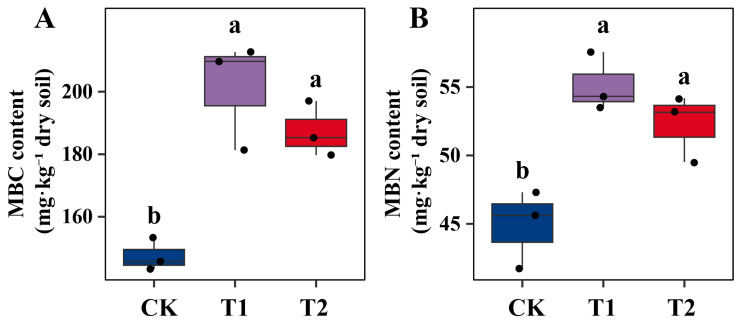
Soil microbial biomass under different treatments in Pb-contaminated soil. Panels (**A**) and (**B**) represent microbial biomass carbon (MBC) and microbial biomass nitrogen (MBN), respectively. CK denotes the control treatment with organic fertilizer only, T1 indicates inoculation with *T. guizhouense* NJAU4742, and T2 indicates inoculation with *B. velezensis* SQR9. Each treatment included three biological replicates. Data are shown as boxplots with individual data points. Different lowercase letters above bars indicate significant differences among treatments at *p* < 0.05 according to one-way ANOVA followed by Tukey’s multiple comparison test.

Microbial inoculation significantly increased soil microbial biomass carbon (MBC) and nitrogen (MBN) under Pb-contaminated conditions (Figure 4). Compared with CK, NJAU4742 raised MBC by 37% and MBN by 23%, while SQR9 increased them by 27% and 16%, respectively (Figure 4A,B). Microbial biomass is widely recognized as a sensitive indicator of soil health, integrating microbial growth, turnover, and nutrient cycling capacity [37,38]. The stronger stimulation by NJAU4742 is consistent with previous findings that *Trichoderma* spp. can enhance rhizosphere microbial activity through root colonization, production of extracellular enzymes (cellulases, chitinases), and secretion of organic acids that mobilize nutrients [39,40]. These compounds are reported to stimulate resident microbial communities and enhance substrate availability, thereby promoting microbial growth and activity [19]. By contrast, *Bacillus* spp. such as SQR9 may contribute to microbial biomass mainly through enhancing nutrient mobilization via organic acid and phosphatase secretion, producing extracellular polymers that improve soil structure and moisture retention, releasing phytohormones and siderophores that stimulate root exudation, and fostering beneficial root–microbe interactions that sustain microbial proliferation [14,15,21]. Thus, while both inoculants increased microbial biomass, their mechanisms of action appear distinct, with *Trichoderma* more directly enhancing nutrient turnover and *Bacillus* providing a protective microenvironment.

**Figure 5:**
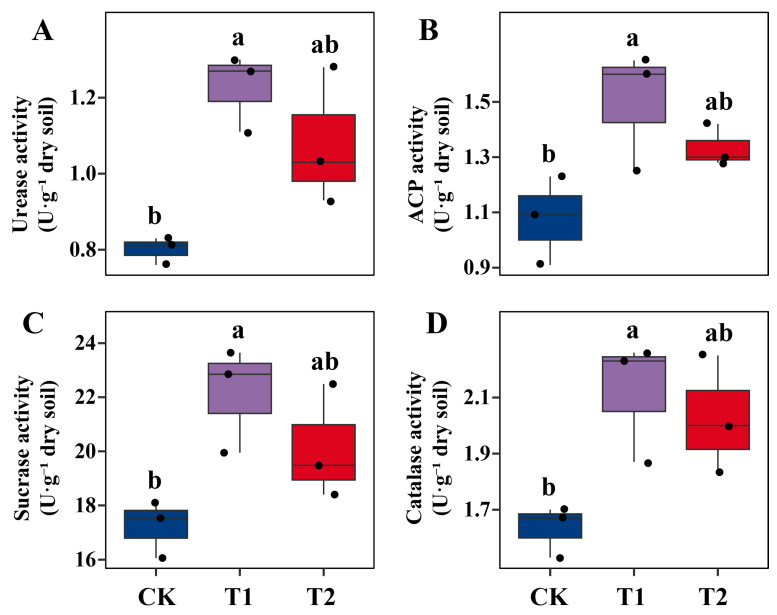
Soil enzyme activities under different treatments in Pb-contaminated soil. Panels (**A**–**D**) represent urease (UE), acid phosphatase (ACP), catalase (CAT), and sucrase (SC), respectively. CK denotes the control treatment with organic fertilizer only, T1 indicates inoculation with *T. guizhouense* NJAU4742, and T2 indicates inoculation with *B. velezensis* SQR9. Each treatment included three biological replicates. Data are shown as boxplots with individual data points. Different lowercase letters above bars indicate significant differences among treatments at *p* < 0.05 according to one-way ANOVA followed by Tukey’s multiple comparison test.

Enzyme activities were also stimulated by microbial treatments, though NJAU4742 consistently produced greater effects than SQR9 (Figure 5A–D). Urease activity is critical for N mineralization, and was increased by more than 50% under NJAU4742, but only modestly under SQR9 (Figure 5A). Acid phosphatase activity which regulates P mobilization from organic forms was also enhanced most strongly by NJAU4742, reflecting improved phosphorus turnover [41] (Figure 5B). Similarly, sucrase activity is an index of C cycling and microbial metabolic activity, and it rose significantly under NJAU4742 (Figure 5C). This aligns with the ecological role of *Trichoderma* as an efficient decomposer that accelerates nutrient turnover [19,42]. Catalase activity showed notable improvement in both inoculants, with NJAU4742 again exerting the strongest effect (Figure 5D). Higher catalase activity indicates improved soil oxidative stability under Pb stress. Heavy metals typically suppress soil enzymes by binding to enzyme active sites or disrupting microbial synthesis pathways [43]. The observed recovery suggests that microbial inoculation mitigated these inhibitory effects. The stronger effect of NJAU4742 on soil enzyme activities is consistent with the known ecological functions of *Trichoderma*, which produces diverse extracellular enzymes that decompose organic matter, mobilize nutrients, and stimulate beneficial soil microbiota [19,42]. By enhancing microbial biomass and enzyme-mediated nutrient cycling, NJAU4742 not only supported plant growth but also improved soil quality and resilience under Pb stress. In contrast, SQR9 showed moderate but consistent improvements in microbial biomass and enzyme activities. Its main contribution may lie in stabilizing the rhizosphere microbial community and protecting roots through biofilm formation and antagonism against pathogens, rather than directly driving soil biochemical turnover [14,15,21]. Improved soil biological activity is particularly important in Pb-contaminated systems, where heavy metal stress often suppresses microbial communities and enzyme functions [44]. By reversing this suppression, microbial fertilizers contribute not only to improved plant performance but also to the long-term ecological restoration of contaminated soils.

### 2.4. Integrative Analysis of Factors Driving Willow Biomass and Pb Accumulation

**Figure 6:**
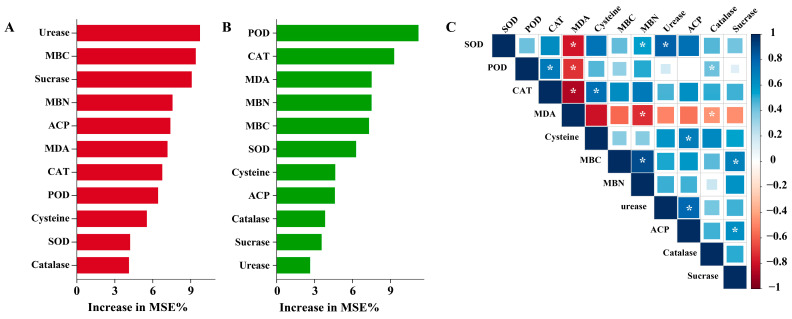
Integrative statistical analysis of plant and soil responses under different treatments in Pb-contaminated soil. Panels (**A**,**B**) show random forest models identifying the relative importance of physiological and soil indices in predicting total biomass (**A**) and total Pb accumulation (**B**). Panel (**C**) presents the Spearman correlation between plant physiological parameters and soil indices. Positive and negative correlations are shown in blue and red, respectively. Significant correlations are marked with * (*p* < 0.05). Abbreviations: SOD, superoxide dismutase; POD, peroxidase; CAT, catalase; MDA, malondialdehyde; MBC, microbial biomass carbon; MBN, microbial biomass nitrogen; ACP, acid phosphatase.

To disentangle the mechanisms by which microbial inoculation influenced willow performance under Pb stress, we conducted random forest analysis using total biomass and total Pb accumulation as explanatory variables. The model explained 86.45% of the total variance, indicating strong predictive reliability. The relative importance of soil and physiological indices was quantified, and their interactions further explored through Spearman correlation analysis (Figure 6). Random forest analysis showed that urease, MBC, and sucrase were the strongest predictors of total biomass, followed by MBN, ACP, and MDA (Figure 6A). This pattern reflects the dominant role of soil nutrient cycling and microbial biomass in sustaining plant growth, whereas antioxidant enzymes contributed to a lesser extent. The strong influence of urease and sucrase highlights that N and C cycling were central to biomass formation under Pb stress. This finding aligns closely with the ecological strategy of *T. guizhouense* NJAU4742, which is known to secrete extracellular enzymes, degrade complex organic matter, and release organic acids that mobilize nutrients [19,42]. By enhancing microbial biomass and accelerating nutrient turnover, NJAU4742 effectively drove the soil-mediated improvements in willow growth observed in this study. Thus, the statistical model supports the conclusion that *Trichoderma* primarily promotes growth by reshaping the nutrient environment of the rhizosphere. In contrast, Pb accumulation was most strongly predicted by antioxidant enzyme activities, particularly POD and CAT, followed by MDA, MBN, and MBC (Figure 6B). This indicates that tolerance to oxidative stress, rather than nutrient availability, was the decisive factor enabling greater Pb sequestration. This pattern is consistent with the functional traits of *B. velezensis* SQR9, which is well known for producing lipopeptides and signaling molecules that prime plant defense responses, leading to stronger activation of antioxidant enzymes and thiol metabolism [45]. Indeed, our physiological data showed that SQR9 markedly increased CAT activity, reduced MDA levels, and enhanced cysteine accumulation, all of which are critical for maintaining cellular integrity and enabling sustained Pb uptake. The random forest output therefore reinforces that *Bacillus* contributes more to Pb accumulation through direct physiological protection than to biomass promotion.

Spearman correlation analysis further clarified the interactions between soil processes and plant physiology (Figure 6C). Negative correlations between antioxidant enzymes (SOD, POD, CAT) and MDA confirmed that microbial treatments alleviated lipid peroxidation by strengthening ROS detoxification pathways, a hallmark effect of SQR9. Positive associations such as CAT–Cys and POD–CAT suggest coordinated activation of enzymatic and thiol-based detoxification mechanisms, again reflecting the influence of *Bacillus*. On the soil side, ACP–sucrase correlations indicated synergistic regulation of P and C cycling, consistent with the activity of *Trichoderma* in enhancing organic matter decomposition and nutrient mobilization [46]. These integrative analyses highlight distinct yet complementary microbial strategies. This division of labor suggests that *Trichoderma* acts as a growth enhancer by optimizing soil function, while *Bacillus* acts as a stress mitigator by reinforcing plant detoxification capacity. Their combined inoculation may therefore offer a synergistic approach, simultaneously maximizing biomass production and Pb uptake.

It should be noted that our study employed organic fertilizer as the baseline control to simulate realistic application conditions. The absence of a no-fertilizer control and a zero-treatment (no Pb, no fertilizer) group represents a limitation of this study, as these would have provided additional clarity regarding the independent effects of organic fertilizer and Pb toxicity. Future research should include these treatments to further disentangle the contributions of soil amendments, microbial inoculants, and Pb stress on plant performance. In addition, this study was conducted under controlled pot conditions using a single clone of *S. suchowensis*. While this design allowed for precise evaluation of microbial inoculant effects, it may not fully represent field-scale dynamics or genetic variability among different willow clones. Future research should therefore include multi-clone and field experiments to further validate the observed effects and assess their stability under natural environmental conditions. Moreover, from a practical perspective, large-scale application of microbial fertilizers in Pb-contaminated soils requires careful consideration of cost, inoculant stability, and field variability. Commercial formulations of *T. guizhouense* and *B. velezensis* are relatively low-cost and have demonstrated stability in agricultural use; however, their persistence and efficacy under heterogeneous field conditions remain to be confirmed. While microbial inoculants can enhance soil remediation efficiency, the potential ecological impacts of introducing large populations of exogenous microbes into disturbed soils should not be overlooked. Future studies should therefore include long-term monitoring and ecological risk assessments to ensure both environmental safety and sustainable remediation outcomes.

## 3. Materials and Methods

### 3.1. Sources of Plant, Microbial Inoculants, and Pb-Contaminated Soil

The plant material used in this study was *S. suchowensis* clonal line P1024, which had been previously identified by our research group as a highly efficient genotype for Pb remediation. This clonal line consistently exhibited superior Pb tolerance, biomass production, and metal accumulation capacity compared with other willow genotypes grown under Pb-contaminated conditions. Two microbial fertilizers were applied as exogenous inoculants: *T. guizhouense* strain NJAU4742 and *B. velezensis* strain SQR9. Both strains were originally isolated and preserved at Nanjing Agricultural University, and were subsequently processed into commercial microbial fertilizer formulations by Huai’an Chaimihe Agricultural Technology Co., Ltd. The activity and viability of the microbial inoculants were confirmed in the laboratory using the plate-counting method. The effective living cell numbers were 8.0 × 10^7^ CFU·g^−1^ for *T. guizhouense* NJAU4742 and 3.0 × 10^9^ CFU·g^−1^ for *B. velezensis* SQR9.

The Pb-contaminated soil used for the pot experiment was collected from the vicinity of a lead-acid battery manufacturing facility. The soil was used in its original state without prior remediation to ensure realistic contamination conditions. Initial soil physicochemical properties were determined before the experiment: total Pb concentration was 17.55 g·kg^−1^, with an available Pb fraction of 4.91 g·kg^−1^. Organic matter content was 34.5 g·kg^−1^, total nitrogen 2.18 g·kg^−1^, available phosphorus 35 g·kg^−1^, and available potassium 0.72 g·kg^−1^.

### 3.2. Experimental Design and Treatments

A total of three treatments were established to evaluate the effects of microbial fertilizers on willow growth and Pb remediation. The control treatment (CK) received only commercial organic fertilizer, while the experimental treatments were supplemented with microbial fertilizers: (T1) *T. guizhouense* NJAU4742 and (T2) *B. velezensis* SQR9. Pb-contaminated soil was homogenized with either the organic fertilizer or the designated microbial fertilizer at an application rate of 2.5 kg·m^−3^. The treated soils were packed into non-woven nursery bags measuring 30 cm in both diameter and height to ensure a uniform growth substrate.

For planting, uniform one-year-old branches of *S. suchowensis* clonal line P1024 were selected. Branches approximately 1.0 cm in basal diameter were cut into 15 cm segments and inserted into the nursery bags, with four cuttings established per bag. A completely randomized design was employed with three treatments and three replicates per treatment. Every replicate consisted of three nursery bags, resulting in a total of nine bags per treatment and 27 bags across the entire experiment.

### 3.3. Determination of Plant Biomass and Pb Content

After six months of growth, whole plants were carefully removed from the nursery bags, and roots were separated from the soil using the soil-washing method to ensure integrity of the root system. Adhering soil particles were rinsed away under running water, followed by repeated washing with deionized water. Excess water was gently removed with absorbent paper. Plants were divided into roots, stems, and leaves. To eliminate Pb ions loosely bound to root surfaces, the roots were immersed in 20 mmol·L^−1^ ethylenediaminetetraacetic acid (EDTA) solution for 15 min, then rinsed again with deionized water. All samples were subjected to a pre-drying step at 105 °C for 30 min to deactivate enzymes, followed by oven-drying at 75 °C to constant weight. Biomass values were expressed as g·plant^−1^.

Dried tissues were ground to a fine powder using a stainless-steel mill, passed through a 0.25 mm sieve, and stored in polyethylene bags prior to digestion. Total Pb concentrations in roots, stems, and leaves were determined separately. Approximately 0.5 g of dried powder was digested with a mixture of concentrated nitric acid and perchloric acid in a digestion block until clear solutions were obtained. After cooling and dilution to a constant volume with deionized water, Pb concentrations were quantified using Inductively Coupled Plasma Mass Spectrometry (ICP-MS) instrument (Thermo, Waltham, MA, USA). Calibration standards were prepared from a Pb stock solution (1000 mg·L^−1^) and diluted to a series of concentrations ranging from 0 to 200 µg·L^−1^. The calibration curve exhibited excellent linearity with an R^2^ value > 0.99. Each QC sample was analyzed in triplicate. Analytical recovery for Pb ranged from 95 to 105%, and relative standard deviation (RSD) was <5%.

### 3.4. Determination of Physiological Parameters

Mature leaves were sampled from each treatment at the end of the experiment for physiological analyses. The activities of superoxide dismutase (SOD), peroxidase (POD), and catalase (CAT) were assayed spectrophotometrically [47]. SOD activity was determined by the nitroblue tetrazolium (NBT) photoreduction method, with one unit defined as the amount of enzyme required to cause 50% inhibition of NBT reduction. POD activity was measured using guaiacol as the substrate by monitoring the increase in absorbance at 470 nm. CAT activity was assessed based on the decomposition rate of H_2_O_2_ at 240 nm.

Malondialdehyde (MDA) content was measured by the thiobarbituric acid (TBA) method [48]. Fresh leaf tissue was homogenized in trichloroacetic acid, reacted with TBA, and absorbance was recorded at 532, 600, and 450 nm to calculate MDA concentration, expressed as nmol·g^−1^ fresh weight. Cysteine (Cys) content was determined using the acid–ninhydrin method [49]. Leaf extracts were reacted with acid–ninhydrin reagent, incubated in boiling water, cooled, and absorbance measured at 560 nm. Concentrations were calculated using a standard curve generated from L-cysteine. All assays were conducted in triplicate.

### 3.5. Soil Sampling and Measurement of Microbial and Enzymatic Indices

Soil samples were collected after complete leaf fall to reflect the end-of-season status of the rhizosphere. Following removal of the top 5 cm, soil was sampled from a depth of 10–20 cm in each treatment plot. Visible plant debris and stones were discarded, and the soil was homogenized, air-dried, gently ground, and passed through a 2 mm sieve for subsequent analyses.

Soil microbial biomass carbon (MBC) and microbial biomass nitrogen (MBN) were quantified using the chloroform fumigation–extraction method [50]. Parallel fumigated and unfumigated soil subsamples were extracted with 0.5 mol·L^−1^ K_2_SO_4_, and extractable C and N concentrations were determined colorimetrically. The differences between fumigated and unfumigated extracts were converted to MBC and MBN using standard correction factors.

To evaluate soil biochemical activity, the activities of four representative enzymes were determined: urease (UE), acid phosphatase (ACP), catalase (CAT), and sucrase (SC). Urease activity was measured by quantifying NH_4_^+^ released from urea hydrolysis [51]; acid phosphatase activity was determined by monitoring p-nitrophenol formation from p-nitrophenyl phosphate [52]; catalase activity was assessed based on the decomposition of H_2_O_2_, with oxygen release as an indicator [53]; and sucrase activity was measured by detecting glucose produced from sucrose hydrolysis [54]. All assays were conducted in triplicate.

### 3.6. Statistical Analyses

All statistical analyses and visualizations were performed using R software (v4.4.1). One-way analysis of variance (ANOVA) followed by Tukey’s multiple comparison test was used to assess differences among treatments, with significance levels set at *p* < 0.05. Random forest (RF) analysis was conducted using the rfPermute package (version 2.5.5) to evaluate the relative contribution of soil and physiological indices to total biomass and total Pb accumulation [55]. Spearman correlation coefficients were calculated using the built-in corr function in R, and significant correlations (*p* < 0.05) were visualized as correlation networks. Graphs were generated with the ggplot2 (version 4.0.0) and corrplot (version 0.95) packages.

## 4. Conclusions

This study demonstrates that microbial inoculation can differentially enhance willow growth and Pb remediation under Pb-contaminated conditions. *T. guizhouense* NJAU4742 promoted plant biomass production, as evidenced by increased soil microbial biomass and higher soil enzyme activities. In contrast, *B. velezensis* SQR9 increased Pb accumulation, supported by stronger antioxidant defenses, reduced lipid peroxidation, and higher cysteine levels. The two inoculants might have complementary functions in soil Pb remediation, with NJAU4742 improving plant growth and soil biological activity, while SQR9 facilitates Pb uptake and tolerance. From a practical perspective, combining both microbial inoculants may provide a synergistic approach to simultaneously increase plant productivity and Pb removal efficiency in contaminated soils. However, future field-scale studies are required to verify these effects under variable soil and environmental conditions, since scalability and site heterogeneity may influence microbial persistence and remediation performance.

## Data Availability

The original contributions presented in this study are included in the article. Further inquiries can be directed to the corresponding authors.
